# Physical Activity Over the Adult Life Course and Risk of Dementia in the Framingham Heart Study

**DOI:** 10.1001/jamanetworkopen.2025.44439

**Published:** 2025-11-19

**Authors:** Francesca R. Marino, Chenglin Lyu, Yuqing Li, Tianyu Liu, Rhoda Au, Phillip H. Hwang

**Affiliations:** 1 Department of Anatomy and Neurobiology, Boston University Chobanian and Avedisian School of Medicine, Boston, Massachusetts; 2 Department of Medicine, Boston University Chobanian and Avedisian School of Medicine, Boston, Massachusetts; 3 Department of Epidemiology, Boston University School of Public Health, Boston, Massachusetts; 4 Department of Neurology, Boston University Chobanian and Avedisian School of Medicine, Boston, Massachusetts; 5 Slone Epidemiology Center, Boston University Chobanian and Avedisian School of Medicine, Boston, Massachusetts; 6 The Framingham Heart Study, Boston University Chobanian and Avedisian School of Medicine, Boston, Massachusetts; 7 Boston University Alzheimer’s Disease and Chronic Traumatic Encephalopathy Center, Department of Neurology, Boston University Chobanian and Avedisian School of Medicine, Boston, Massachusetts

## Abstract

**Importance:**

Being physically active is protective against dementia. Yet, it is unknown when during the adult life course physical activity is most associated with dementia risk.

**Objective:**

To determine whether higher physical activity levels in early adult life, midlife, or late life are associated with lower risk of all-cause or Alzheimer disease (AD) dementia.

**Design, Setting, and Participants:**

This prospective cohort study used data from the Framingham Heart Study Offspring cohort. The offspring of participants in the original Framingham Heart Study cohort who were dementia free and had physical activity measured at baseline (early adult life [1979-1983], midlife [1987-1991], or late life [1998-2001]) were followed up for a mean (SD) of 37.2 (7.1), 25.9 (8.5), or 14.5 (6.6) years for the development of incident all-cause or AD dementia until December 31, 2023.

**Exposure:**

Physical activity was self-reported using the physical activity index, a composite score weighted by hours spent sleeping and in sedentary, slight, moderate, or heavy activities. Physical activity was divided into quintiles (Q).

**Main Outcome and Measure:**

All-cause and AD dementia were classified by expert consensus based on established diagnostic criteria.

**Results:**

This study included 1526 early adult–life (mean [SD] age, 36.7 [4.7] years; 821 [53.8%] female), 1943 midlife (mean [SD] age, 54.0 [5.8] years; 1010 [52.0%] female), and 885 late-life (mean [SD] age, 71.0 [4.5] years; 473 [53.4%] female) participants. There were 567 cases of incident all-cause dementia during follow-up. Higher levels of midlife and late-life physical activity were associated with lower risk of all-cause dementia. Midlife and late-life physical activity levels in Q4 or Q5 were associated with lower risk of all-cause dementia compared with Q1 (midlife Q4: HR, 0.60; 95% CI, 0.41-0.89; midlife Q5: HR, 0.59; 95% CI, 0.40-0.88; late-life Q4: HR, 0.64; 95% CI, 0.42-1.00; late-life Q5: HR, 0.55; 95% CI, 0.35-0.87). There were no associations between early adult–life physical activity and dementia risk. Findings were similar for incident AD (369 cases).

**Conclusions and Relevance:**

In this cohort study of adults in the Framingham Heart Study Offspring cohort, higher levels of midlife and late-life physical activity were associated with similar reductions in risk of all-cause and AD dementia. These findings may inform future efforts to delay or prevent dementia through timing interventions during the most relevant stages of the adult life course.

## Key Points

**Question:**

When during the adult life course is physical activity most associated with risk of incident dementia?

**Findings:**

In this cohort study of 1526 early adult–life, 1943 midlife, and 855 late-life participants in the Framingham Heart Study, individuals with the highest levels of physical activity at midlife and late life had 41% and 45% lower risk of all-cause dementia, respectively, compared with those with the lowest levels of physical activity, a statistically significant difference. Early adult–life physical activity was not associated with dementia risk.

**Meaning:**

These findings suggest that timing efforts to promote physical activity during midlife or late life may be warranted to help delay or prevent dementia.

## Teaser

This cohort study uses data from the Framingham Heart Study to evaluate whether higher physical activity levels in early adult life, midlife, or late life are associated with lower risk of all-cause or Alzheimer disease dementia.

## Introduction

There is a critical need for effective treatments and preventive strategies for dementia.^1^ Pharmacologic treatments for Alzheimer disease (AD) exhibit some efficacy in slowing cognitive decline; however, these drugs are expensive and can have adverse side effects.^2^ Thus, there is growing interest in better understanding lifestyle risk factors for preventing or slowing the decline to dementia. The 2024 Lancet Commission estimates that up to 45% of dementia cases could be prevented by modifying 14 risk factors during different times in the life course, one of which is midlife physical activity.^2^ This framework recognizes the importance of maintaining health and managing risk factors earlier in life. However, it remains unclear exactly when the critical periods for these risk factors are.

Physical activity may be an important modifiable lifestyle factor that can delay or possibly prevent the onset of dementia. To date, most studies have focused on either midlife or late-life physical activity and found positive associations with brain structure or connectivity,^3,4,5,6,7,8,9^ cognitive function,^10,11,12,13^ and dementia risk.^13,14,15,16,17,18,19,20,21,22^ Physical activity earlier in life is also linked with better cognitive function^23,24,25,26^ and lower dementia risk.^16^ However, many studies examining early life physical activity rely on older adult participants to recall their physical activity levels from earlier in life.^12,16,26,27,28^ This likely results in measurement error, as older adults, particularly those experiencing cognitive impairment, may incorrectly recall their physical activity earlier in life.^29^ This may also lead to reverse causation bias due the bidirectionality between physical activity and cognitive function.^30^ Therefore, more research is needed to determine whether physical activity exerts a stronger influence on dementia risk during different periods of the adult life course. This has important implications for intervention planning and public health promotion.

To address this gap, the current study aims to leverage data from the Framingham Heart Study (FHS) to evaluate associations between self-reported physical activity measured over the adult life course and risk of dementia. Specifically, this study will identify whether physical activity during early adult life, midlife, or late-life is more associated with dementia risk. We hypothesize that higher physical activity in early adult life, midlife, and late life is associated with lower risk of dementia, where midlife physical activity is most associated with on dementia risk.

## Methods

### Study Population

**Figure 1.**
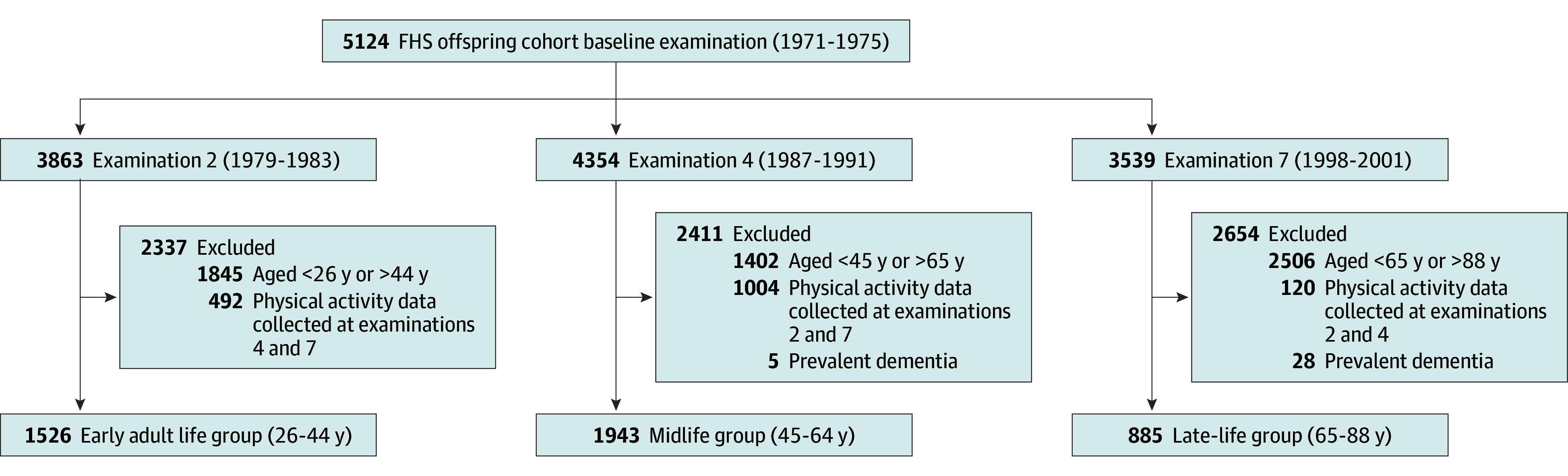
Flow Diagram of Study Sample FHS indicates Framingham Heart Study.

Initiated in 1948, FHS is a population-based cohort with regular health examinations.^31^ The present study used data from the FHS Offspring (Generation 2) cohort, which began in 1971 to 1975 with 5124 participants who were aged 5 to 70 years and had, on average, health examinations every 4 to 8 years.^32^ To study the association between physical activity across the adult life course and dementia risk, analyses were based on 1526 participants at examination 2 (1979-1983) who were aged 26 to 44 years (early adult–life group), 1943 participants at examination 4 (1987-1991) who were aged 45 to 64 years (midlife group), and 885 participants at examination 7 (1998-2001) who were aged 65 to 88 years (late-life group). All participants were included in the sample based on being dementia-free and having information on physical activity collected only at their respective baseline examination (Figure 1). This study followed the Strengthening the Reporting of Observational Studies in Epidemiology (STROBE) reporting guidelines for cohort studies. Informed consent was obtained from all study participants, and the study protocol was approved by the institutional review board of the Boston University Medical Campus.

### Physical Activity

Physical activity was assessed using the physical activity index (PAI), which is a composite score constructed by weighting each hour in a typical day based on activity levels (based on oxygen consumption or metabolic equivalents) and summing up these weighted hours over a 24-hour period. Participants reported the number of hours in a typical day spent sleeping (weighting factor [WF] = 1.0), and in sedentary (WF = 1.1), slight (WF = 1.5), moderate (WF = 2.4), and heavy activities (WF = 5.0).^33^ Validity of these variables, by correlation of comparable physical activity questionnaires to accelerometer-derived physical activity measures, has been shown to be fair, but it may be that this correlation is as high as can be expected from a short physical activity questionnaire.^34,35^ As such, higher PAI scores are interpreted as higher physical activity levels. PAI scores were divided into age group-specific quintiles (Q; eg, early adult life, midlife, and late life), from lowest (Q1) to highest (Q5), with Q1 serving as the reference group.

### Dementia Ascertainment

Participants suspected of cognitive impairment were classified by expert consensus, including a team with at least 1 neurologist and 1 neuropsychologist, as to whether they have dementia.^36^ For this study, we followed up the study sample for the development of incident dementia until December 31, 2023. Diagnosis of all-cause dementia was based on the fourth edition of the *Diagnostic and Statistical Manual of Mental Disorders*.^37^ Diagnosis of AD dementia was based on the National Institute of Neurological and Communicative Disorders and Stroke and the Alzheimer Disease and Related Disorders Association criteria.^38^ Detailed procedures for the diagnosis of dementia within FHS are reported elsewhere.^36^

### Covariates

Potential confounders included baseline age (years), sex (female vs male), education (high school but did not graduate vs high school graduate vs some college vs college graduate), body mass index (BMI; calculated as weight in kilograms divided by height in meters squared), current smoking status (yes vs no), hypertension, diabetes, hyperlipidemia, and apolipoprotein E (*APOE*) ε4 carrier status (at least 1 ε4 allele vs no ε4 allele).

Hypertension was defined as having systolic/diastolic blood pressure of 130/80 mmHg or higher, or the use of antihypertensive medication for treating high blood pressure.^39^ Diabetes was defined as having nonfasted blood glucose of 200 mg/dL or higher, fasted blood glucose of 126 mg/dL or higher, or the use of insulin or hypoglycemic medication to lower blood glucose.^40^ Hyperlipidemia was defined as having total cholesterol higher than 200 mg/dL, or the use of cholesterol medications.

### Statistical Analysis

Based on the baseline examination for each sample (eg, early adult life group, midlife group, and late-life group), we provided descriptive statistics: mean (SD) for continuous variables or count and percentage for categorical variables. Survival analysis using Cox regression models were used to compute hazard ratios (HRs) and 95% CIs to estimate the risk of all-cause dementia associated with each increasing Q of physical activity, with the lowest Q of PAI as a reference group (Q1), for the early adult life, midlife, and late-late groups. The proportional hazards assumption was checked using statistical tests and graphical diagnostics based on the scaled Schoenfeld residuals. Participants were followed up from each baseline examination until the occurrence of dementia diagnosis or death. They were censored at the dates on which these events occurred. Those who did not develop any of the previously mentioned events were followed up until the end of the study (December 31, 2023), at which time they were censored. All models were adjusted for age, sex, education, BMI, current smoking status, hypertension, diabetes, hyperlipidemia, and *APOE* ε4 carrier status. As a sensitivity analysis, we also categorized PAI scores into tertiles and quartiles to estimate the risk of all-cause dementia associated with each increasing tertile and quartile of physical activity, respectively, for the early adult–life, midlife, and late-life groups.

In secondary analyses, models for each age group were stratified by *APOE* ε4 carrier status to evaluate whether being an ε4 carrier modifies the association between physical activity and incident all-cause dementia. In addition, since the measure of physical activity used in our primary analysis (eg, PAI) is a composite measure of different activity levels, we assessed associations between physical activity intensity (eg, slight, moderate, or heavy) and risk of all-cause dementia for each age group. Age group-specific Qs based on the number of hours reported in each physical activity intensity were calculated. Finally, we evaluated associations between physical activity levels with AD dementia as the outcome in the analysis for each age group. The significance level was set to .05. All analyses were performed using Stata version 17.0 (StataCorp).

## Results

### Sample Characteristics

This study included 1526 early adult–life (mean [SD] age, 36.7 [4.7] years; 821 [53.8%] female), 1943 midlife (mean [SD] age, 54.0 [5.8] years; 1010 [52.0%] female), and 885 late-life (mean [SD] age, 71.0 [4.5] years; 473 [53.4%] female) participants. Late-life participants tended to have lower education, higher BMI, lower prevalence of current smoking, and higher prevalence of hypertension, diabetes, and hypercholesteremia than younger participants (Table 1).

**Table 1.** Sample Characteristics of Early Adult–Life, Midlife, and Late-Late Participants From the Framingham Heart Study Offspring Cohort

Characteristic	Participants, No. (%)		
	Early adult life (aged 26-44 y) (n = 1526)	Midlife (aged 45-64 y) (n = 1943)	Late-life (aged 65-88 y) (n = 885)
Age, mean (SD), y	36.7 (4.7)	54.0 (5.8)	71.0 (4.5)
Sex			
Female	821 (53.8)	1010 (52.0)	473 (53.4)
Male	705 (46.2)	933 (48.0)	412 (46.6)
Educational attainment			
High school, did not graduate	46 (3.0)	140 (7.2)	72 (8.0)
High school graduate	418 (27.4)	643 (33.1)	335 (37.9)
Some college	473 (31.0)	573 (29.5)	228 (25.8)
College graduate	589 (38.6)	587 (30.2)	250 (28.3)
Body mass index, mean (SD)^a^	25.0 (4.4)	27.2 (4.8)	28.0 (4.8)
Current smoker	601 (39.4)	474 (24.4)	62 (7.0)
Hypertension	565 (37.0)	1253 (64.5)	646 (73.0)
Diabetes	6 (0.4)	117 (6.0)	147 (16.6)
Hypercholesteremia	586 (38.4)	1144 (58.9)	599 (67.7)
*APOE* ε4 allele carrier	311 (20.4)	443 (22.8)	193 (21.8)
Follow up time, mean (SD), y	37.2 (7.1)	25.9 (8.5)	14.5 (6.6)
Physical activity index, mean (range)			
Overall	34.8 (26.3-64.2)	36.9 (25.6-74.5)	38.4 (25.2-64.6)
1st Quintile (lowest)	29.2 (26.3-30.4)	29.6 (25.6-31.1)	30.7 (25.2-33.1)
2nd Quintile	31.4 (30.5-32.3)	32.5 (31.2-33.9)	34.5 (33.2-35.7)
3rd Quintile	33.4 (32.4-34.4)	35.2 (34.0-36.8)	37.4 (35.8-39.2)
4th Quintile	36.0 (34.5-38.4)	39.0 (36.9-41.4)	41.0 (39.3-43.0)
5th Quintile (highest)	43.8 (38.5-64.2)	47.9 (41.5-74.5)	48.0 (43.1-64.6)

Abbreviation: *APOE*, apolipoprotein E.

^a^ Calculated as weight in kilograms divided by height in meters squared.

**Figure 2.**
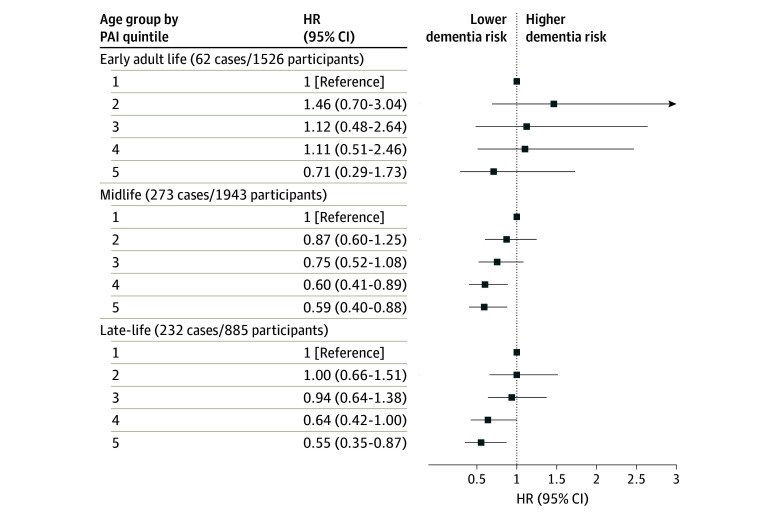
Forest Plots of Associations Between Physical Activity in Early Adult Life, Midlife, and Late-Life Groups and Incident All-Cause Dementia Adjusted for age, sex, education, body mass index, smoking status, hypertension, diabetes, hyperlipidemia, and *APOE* e4 carrier status. HR indicates hazard ratio; PAI, physical activity index.

Overall, 567 participants transitioned to all-cause dementia over follow-up, including 62 early adult–life (4%), 273 midlife (14%), and 232 late-life participants (26%) (Figure 2). Participants in each ascending age group were followed for a mean (SD) of 37.2 (7.1), 25.9 (8.5), or 14.5 (6.6) years (Table 1). There were 320 deaths and 30 losses to follow-up among participants in the early adult life group, 777 and 58 among the midlife group, and 433 and 44 among the late-life group, respectively. Participants with lower PAI had higher mortality rates than those with higher PAI across all age groups (eTable 1 in Supplement 1).

### Physical Activity and Risk of Incident All-Cause Dementia

Higher physical activity in midlife and late life, but not early adult life, was associated with lower risk of incident all-cause dementia. For both midlife and late life, participants with physical activity in Q4 or Q5 had lower risk of dementia compared with those in Q1 after covariate adjustment (midlife Q4: HR, 0.60; 95% CI, 0.41-0.89; midlife Q5: HR, 0.59; 95% CI, 0.40-0.88; late-life Q4: HR, 0.64; 95% CI, 0.42-1.00; late-life Q5: HR, 0.55; 95% CI, 0.35-0.87). Compared with Q1, midlife and late life physical activity in Q2 or Q3 was not associated with dementia risk. Early adult life physical activity was not associated with risk of dementia (Figure 2). From the sensitivity analysis, categorizing physical activity into tertiles or quartiles did not substantially change the results compared with categorizing physical activity into Qs as was done in the primary analysis (eTables 2 and 3 in Supplement 1).

### Differences in Physical Activity and Dementia Associations by *APOE* ε4 Carrier Status

*APOE* ε4 status modified associations between midlife physical activity and incident all-cause dementia risk (*P* for interaction = .04). Among noncarriers, midlife physical activity in Q4 or Q5 was associated with 49% and 59% lower risk of dementia compared with Q1, respectively (Q4: HR, 0.51; 95% CI, 0.31-0.83; Q5: HR, 0.41; 95% CI, 0.24-0.70). Additionally, late-life physical activity in Q5 was associated with 46% lower risk of dementia after covariate adjustment (HR, 0.54; 95% CI, 0.31-0.95) (Table 2). *APOE* ε4 carriers with the late-life physical activity in Q5 also had 66% lower risk of dementia compared with Q1 (HR, 0.34; 95% CI, 0.12-0.94) (Table 2).

**Table 2.** Associations Between Physical Activity and Risk of Incident All-Cause Dementia Among Early Adult–Life, Midlife, and Late-Life Adults by *APOE* ε4 Carrier Status

Physical activity index quintile	Early adult life^a^				Midlife^a^				Late-life^a^			
	Crude model		Adjusted model^b^		Crude model		Adjusted model^b^		Crude model		Adjusted model^b^	
	HR (95% CI)	*P* value	HR (95% CI)	*P* value	HR (95% CI)	*P* value	HR (95% CI)	*P* value	HR (95% CI)	*P* value	HR (95% CI)	*P* value
ε4 Carrier^c^												
1st (lowest)	1.00 [Reference]	NA	1.00 [Reference]	NA	1.00 [Reference]	NA	1.00 [Reference]	NA	1.00 [Reference]	NA	1.00 [Reference]	
2nd	2.00 (0.69-5.79)	.20	2.29 (0.78-6.78)	.13	1.32 (0.71-2.44)	.38	1.65 (0.82-3.35)	.16	1.72 (0.88-3.35)	.11	1.56 (0.77-3.16)	.22
3rd	0.90 (0.24-3.45)	.88	0.90 (0.23-3.60)	.89	1.17 (0.63-2.20)	.61	1.13 (0.55-2.34)	.73	1.10 (0.58-2.10)	.77	1.07 (0.56-2.04)	.83
4th	0.74 (0.20-2.82)	.66	0.69 (0.15-3.17)	.64	0.90 (0.46-1.78)	.76	0.93 (0.46-1.87)	.84	0.66 (0.29-1.50)	.32	0.52 (0.23-1.16)	.11
5th (highest)	0.38 (0.08-1.85)	.23	0.35 (0.07-1.82)	.21	0.87 (0.45-1.69)	.68	0.82 (0.39-1.69)	.59	0.39 (0.15-1.04)	.06	0.34 (0.12-0.94)	.04
Non-ε4 carrier^d^												
1st (lowest)	1.00 [Reference]	NA	1.00 [Reference]	NA	1.00 [Reference]	NA	1.00 [Reference]	NA	1.00 [Reference]	NA	1.00 [Reference]	
2nd	2.00 (0.62-6.50)	.25	2.21 (0.65-7.51)	.20	0.72 (0.47-1.12)	.14	0.70 (0.43-1.13)	.14	0.76 (0.48-1.21)	.25	0.82 (0.51-1.31)	.40
3rd	1.91 (0.58-6.30)	.29	2.04 (0.63-6.64)	.23	0.69 (0.44-1.07)	.10	0.69 (0.43-1.11)	.12	0.65 (0.41-1.03)	.07	0.79 (0.48-1.32)	.37
4th	0.95 (0.24-3.79)	.94	1.07 (0.27-4.26)	.93	0.59 (0.37-0.93)	.02	0.51 (0.31-0.83)	.01	0.60 (0.37-0.96)	.03	0.74 (0.45-1.21)	.23
5th (highest)	0.93 (0.23-3.69)	.91	0.97 (0.24-3.92)	.97	0.48 (0.29-0.78)	<.01	0.41 (0.24-0.70)	<.01	0.53 (0.31-0.90)	.02	0.54 (0.31-0.95)	.03

Abbreviation: HR, hazard ratio.

^a^ *P* value for interaction between physical activity and *APOE* ε4 carrier status reflects physical activity operationalized as a dichotomous variable (higher physical activity index [quartile 2 to quartile 5] vs low physical activity index [quartile 1]), and is based on the adjusted model. Early adult life *P* = .68; midlife *P* = .04; and late life *P* = .62.

^b^ Adjusted for age, sex, education, body mass index, smoking status, hypertension, diabetes, and hyperlipidemia.

^c^ ε4 Carrier only composed of 311 participants and 26 all-cause dementia cases in early adult life, 1500 participants and 171 all-cause dementia cases in midlife, and 692 participants and 159 all-cause dementia cases in late life.

^d^ ε4 Noncarrier only composed of 1215 participants and 29 all-cause dementia cases in early adult life, 443 participants and 171 all-cause dementia cases in midlife, and 193 participants and 72 all-cause dementia cases in late life.

### Physical Activity Intensity and Risk of Incident All-Cause Dementia

Higher moderate or heavy physical activity in midlife, but not early adult or late life, was associated with lower risk of incident all-cause dementia. Moderate midlife physical activity in Q4 or Q5 was associated with 35% and 38% lower risk of dementia compared with Q1 (Q4: HR, 0.65; 95% CI, 0.44-0.97; Q5: HR, 0.62; 95% CI, 0.42-0.92). Additionally, heavy physical activity in Q5 was associated with 34% lower risk of dementia compared with Q1 (HR, 0.66; 95% CI, 0.44-1.00) (eTable 4 in Supplement 1). Slight physical activity was not associated with dementia risk (eTable 4 in Supplement 1).

### Physical Activity and Risk of Incident Alzheimer Dementia

Findings were similar when evaluating associations between physical activity and risk of incident AD dementia. There were 35 cases of incident AD dementia among early adult–life, 178 among midlife, and 156 among late-life participants. For both midlife and late-life, physical activity in Q5 was associated with lower risk of AD dementia compared with Q1 (midlife: HR, 0.55; 95% CI, 0.33-0.94; late-life: HR, 0.53; 95% CI, 0.29-0.95). (eTable 5 in Supplement 1).

Associations between midlife physical activity and incident AD dementia risk differed by *APOE* ε4 status (*P* for interaction = .01). Findings were robust for middle and late-life physical activity and AD dementia risk among noncarriers (midlife Q4: HR, 0.45; 95% CI, 0.24-0.85; midlife Q5: HR, 0.35; 95% CI, 0.18-0.70; late-life Q5: HR, 0.43; 95% CI, 0.21-0.89). Findings for late-life physical activity and AD dementia risk were not statistically significant among ε4 carriers (eTable 6 in Supplement 1).

## Discussion

Among community-dwelling adults in the FHS Offspring cohort, we found that higher physical activity levels in midlife and late life were associated with lower risk of both all-cause and AD dementia. Findings in midlife were associated with moderate or heavy physical activity intensities, whereas late-life findings did not differ across physical activity intensity groups. The benefits of midlife or late-life physical activity also differed by *APOE* ε4 status. Overall, these results support the importance of midlife and late-life physical activity for dementia risk reduction, highlighting key stages of the adult life course for intervention planning and public health promotion. Future studies with longer follow-up periods are needed to clarify the role of early adult life physical activity for dementia risk mitigation.

To appropriately plan interventions and preventive strategies, it is important to understand when during the adult life course physical activity is most associated with dementia risk. Our findings suggest that higher midlife and late-life physical activity are associated with similar reductions in all-cause and AD dementia risk. This aligns with prior studies finding associations between higher self-reported^20,21^ or objectively measured physical activity^15^ in midlife or late-life with lower all-cause dementia risk. Similar associations are also reported with AD dementia risk.^16,19,20,41^ However, there are inconsistencies across studies, where some have only found associations with midlife^17^ or late-life^13,42^ physical activity. When evaluating specific physical activity intensities, we found that moderate or heavy physical activity in midlife was associated with lower dementia risk, but that risk did not differ across intensities of late-life physical activity. This is consistent with previous work showing that more moderate or vigorous physical activity in midlife is associated with reduced dementia risk.^18,19,22^ While some studies report benefits of higher intensity physical activity in late-life,^43^ emerging evidence suggests that even light intensity physical activity is beneficial for cognitive health among older adults.^44^ This study builds on previous findings to support moderate or heavy midlife physical activity and any late-life physical activity as possible interventions for dementia risk reduction.

In this study, early adult life physical activity was not associated with dementia risk. This aligns with prior prospective studies that did not find associations between physical activity measured in early adulthood and risk of incident dementia.^17^ However, other studies have found that higher physical activity in early adulthood is associated with lower odds of subjective cognitive impairment^28^ or AD risk^16^ later in life. These differences in findings could be from studies asking older adult participants to recall their previous activity levels from earlier in life,^16,28^ which could lead to possible recall bias or reverse causation bias.^29,30^ It is also possible that we did not detect associations with early adult life physical activity due to the smaller number of dementia cases in this age group. Indeed, participants in the early adult life group had fewer dementia cases and a shorter etiologically relevant window of exposure, limiting our ability to draw robust conclusions about associations between early adult life physical activity and dementia risk. Nonetheless, there are still important benefits of early adult life physical activity including reduced cardiovascular risk^45^ and better mental health,^46,47^ both of which relate to future dementia risk.^2^ Additional studies with larger, more diverse samples and longer follow-up times are needed to observe potentially significant associations between physical activity in early adult life and dementia risk.

There are several possible mechanisms through which physical activity is thought to lower the risk of all-cause or AD dementia. First, physical activity may directly lower AD-related neuropathology, possibly through slowing amyloid-β (Aβ) production, accelerating Aβ clearance, or altering τ phosphorylation.^48,49^ Second, physical activity likely improves brain structure and function through its upregulation of brain neurotrophins^48,49^ that promote hippocampal neurogenesis, angiogenesis, synaptogenesis, cerebral blood flow, endothelial function, and small vessel integrity.^48^ Third, physical activity is associated with acute anti-inflammatory effects immediately after activity, as well as sustained effects via reductions in abdominal and visceral fat.^48^ Chronic inflammation is also related to worse immune function, likely through decreasing levels of interleukin-6 and Aβ deposition.^48^ Fourth, physical activity can help counter impairments in glucose metabolism, which can cause vascular dysfunction, through improving insulin signaling, glucose transport, and insulin sensitivity.^48^ Finally, physical activity may exert vascular benefits through reducing stress.^49^ Overall, physical activity at any age is thought to facilitate cognitive reserve and delay late-life cognitive impairment.^50^ However, it has yet to be determined whether these mechanisms are mutually exclusive or if they act together at the same times during the life course. Future studies should examine whether different biological mechanisms exist during middle or late life that explain associations between physical activity and dementia.

In this study, higher midlife physical activity was only associated with lower dementia risk among *APOE* ε4 allele noncarriers, whereas late-life physical activity was associated with dementia risk among both ε4 carriers and noncarriers. It is generally thought that associations between physical activity and cognitive impairment^43^ or dementia^42^ are greater among noncarriers. Physical activity and *APOE* ε4 exert opposite effects on many neuropathological changes including, but not limited to, Aβ deposition, brain neurotrophic factors, and cerebrovascular function.^48^ Physical activity may partly, but not fully, offset or delay these *APOE* ε4-related changes,^48^ which could be a potential reason why findings were observed in middle and late life for noncarriers but only in late life among carriers. Future studies are needed to confirm whether physical activity levels during certain periods of the adult life course confer differential dementia risk reduction by *APOE* ε4 status.

### Limitations and Strengths

This study has several limitations. First, participants in the FHS Offspring cohort are primarily of European backgrounds. Generalizability to other racial and ethnic populations may be limited, although efforts are under way to enroll participants from move diverse populations. Second, we may have been underpowered to detect associations with early adult life physical activity due to the small number of dementia cases in this age group. Third, due to the timing of physical activity measurements, we were unable to evaluate associations earlier in the life course (eg, adolescence) or earlier in adulthood for the midlife and late life age groups. Fourth, we were unable to evaluate associations with dementia subtypes aside from AD dementia. Fifth, it is possible that physical activity levels were misclassified, as we used a self-reported measure at 1 time point. Future studies should collect repeated measures of physical activity or use objective physical activity measures (eg, accelerometers) to remedy this potential misclassification. The PAI is also limited in its clinical interpretability as compared with other physical activity metrics (eg, steps). Sixth, some participants were censored due to death or loss to follow-up. We found that mortality rates were higher among participants with lower vs higher PAI and of older vs younger ages. We expect this would lead to an underestimation of the true association between PAI and dementia. Given the small proportion of loss to follow-up in this study, we do not expect bias due to differential loss to follow-up. Additionally, reverse causation between cognitive function and physical activity is a potential concern in the late-life age group. However, this concern is attenuated by the long follow-up time (mean 14.5 years) and exclusion of prevalent dementia cases at baseline. There are also many strengths of this study. First, we applied a life course framework when evaluating the benefits of physical activity for dementia risk reduction by examining physical activity levels during different periods of the adult life course. Second, we employed a prospective study design and had a long follow-up period for each age group. Third, dementia and AD status were adjudicated by consensus conference.^36^ Additionally, FHS cohorts are well-characterized, and we had information available on important demographic, lifestyle, and medical covariates.

## Conclusions

In this cohort study of adults in the FHS Offspring cohort, we found that higher physical activity levels in midlife and late-life were associated with similar reductions in all-cause dementia and AD dementia risk. This study is among the first to evaluate the potential critical periods for physical activity in association with dementia risk. These findings may inform future efforts to delay or prevent dementia through timing interventions and public health promotion efforts during the most relevant stages of the adult life course.

## Notes

Supplement 1. **eTable 1.** Proportion of Deaths and Losses to Follow-Up Overall and by Physical Activity Levels Among Early Adult Life, Midlife, and Late-Life Adults **eTable 2. **Associations Between Tertile-Based Physical Activity Levels and Risk of Incident All-Cause Dementia Among Early Adult Life, Midlife, and Late-Life Adults **eTable 3. **Associations Between Quartile-Based Physical Activity Levels and Risk of Incident All-Cause Dementia Among Early Adult Life, Midlife, and Late-Life Adults **eTable 4.** Associations Between Physical Activity Intensity (Slight, Moderate, Heavy) Levels and Risk of Incident All-Cause Dementia Among Early Adult Life, Midlife, and Late-Life Adults **eTable 5. **Associations Between Physical Activity and Risk of Incident Alzheimer’s Disease Dementia Among Early Adult Life, Midlife, and Late-Life Adults **eTable 6.** Associations Between Physical Activity and Risk of Incident Alzheimer’s Disease Dementia Among Early Adult Life, Midlife, and Late-Life Adults by *APOE* ε4 Carrier Status

Supplement 2.
Data Sharing Statement
