# Supplementary material for: Physical Activity Over the Adult Life Course and Risk of Dementia in the Framingham Heart Study
Source: JAMA Netw Open. 2025 Nov 19;8(11):e2544439. doi: 10.1001/jamanetworkopen.2025.44439 (PMC12631490; doi:10.1001/jamanetworkopen.2025.44439)
Supplement: Supplement 1. — eTable 1. Proportion of Deaths and Losses to Follow-Up Overall and by Physical Activity Levels Among Early Adult Life, Midlife, and Late-Life Adults eTable 2. Associations Between Tertile-Based Physical Activity Levels and Risk of Incident All-Cause Dementia Among Early Adult Life, Midlife, and Late-Life Adults eTable 3. Associations Between Quartile-Based Physical Activity Levels and Risk of Incident All-Cause Dementia Among Early Adult Life, Midlife, and Late-Life Adults eTable 4. Associations Between Physical Activity Intensity (Slight, Moderate, Heavy) Levels and Risk of Incident All-Cause Dementia Among Early Adult Life, Midlife, and Late-Life Adults eTable 5. Associations Between Physical Activity and Risk of Incident Alzheimer’s Disease Dementia Among Early Adult Life, Midlife, and Late-Life Adults eTable 6. Associations Between Physical Activity and Risk of Incident Alzheimer’s Disease Dementia Among Early Adult Life, Midlife, and Late-Life Adults by APOE ε4 Carrier Status [file jamanetwopen-e2544439-s001.pdf]

Supplemental Online Content

Marino FR, Lyu C, Li Y, Liu T, Au R, Hwang PH. Physical activity over the adult life course and risk of dementia in the Framingham Heart Study. *JAMA Netw Open.* 2025;8(11):e2544439. doi:10.1001/jamanetworkopen.2025.44439

eTable 1. Proportion of Deaths and Losses to Follow-Up Overall and by Physical Activity Levels Among Early Adult Life, Midlife, and Late-Life Adults

eTable 2. Associations Between Tertile-Based Physical Activity Levels and Risk of Incident All-Cause Dementia Among Early Adult Life, Midlife, and Late-Life Adults

eTable 3. Associations Between Quartile-Based Physical Activity Levels and Risk of Incident All-Cause Dementia Among Early Adult Life, Midlife, and Late-Life Adults

eTable 4. Associations Between Physical Activity Intensity (Slight, Moderate, Heavy) Levels and Risk of Incident All-Cause Dementia Among Early Adult Life, Midlife, and Late-Life Adults

eTable 5. Associations Between Physical Activity and Risk of Incident Alzheimer's Disease Dementia Among Early Adult Life, Midlife, and Late-Life Adults

eTable 6. Associations Between Physical Activity and Risk of Incident Alzheimer's Disease Dementia Among Early Adult Life, Midlife, and Late-Life Adults by *APOE* ε4 Carrier Status

This supplemental material has been provided by the authors to give readers additional information about their work.

© 2025 Marino FR et al. *JAMA Network Open*.

© 2025 Marino FR et al. *JAMA Network Open*.

eTable 1. Proportion of Deaths and Losses to Follow-Up Overall and by Physical Activity Levels Among Early Adult Life, Midlife, and Late-Life Adults

**Physical Activity Index**	**Early adult life (n=1,526)**	**Midlife (n=1,943)**	**Late-life (n=885)**
1st quintile (lowest) #deaths/# participants (%)	75/305 (24%)	165/388 (42%)	99/177 (56%)
2nd quintile #deaths/# participants (%)	65/305 (21%)	162/389 (42%)	95/176 (54%)
3rd quintile #deaths/# participants (%)	62/304 (20%)	159/388 (41%)	87/177 (49%)
4th quintile #deaths/# participants (%)	60/305 (20%)	147/389 (38%)	78/178 (44%)
5th quintile #deaths/# participants (%)	58/307 (19%)	144/389 (37%)	74/177 (42%)
**Total #deaths/Total #participants**	**320/1526 (21%)**	**777/1943 (40%)**	**433/885 (49%)**
**Total #loss to follow-up/Total #participants**	**30/1526 (2%)**	**58/1943 (3%)**	**44/885 (5%)**

© 2025 Marino FR et al. *JAMA Network Open*.

eTable 2. Associations Between Tertile-Based Physical Activity Levels and Risk of Incident All-Cause Dementia Among Early Adult Life, Midlife, and Late-Life Adults

	Early adult life		Midlife		Late-life	
**Physical activity index**	**Hazard ratio[a] (95% confidence interval)**	***P***	**Hazard ratio[a] (95% confidence interval)**	***P***	**Hazard ratio[a] (95% confidence interval)**	***P***
Tertiles						
1st tertile (lowest)	1.00 (Reference)		1.00 (Reference)		1.00 (Reference)	
2nd tertile	0.91 (0.50, 1.64)	0.76	0.96 (0.72, 1.28)	0.80	0.98 (0.71, 1.35)	0.91
3rd tertile (highest)	0.74 (0.40, 1.38)	0.34	0.90 (0.67, 1.20)	0.46	0.90 (0.65, 1.24)	0.51

P = p-value

[a]Adjusted for age, sex, education, body mass index, smoking status, hypertension, diabetes, hyperlipidemia, *APOE* ε4 carrier status

© 2025 Marino FR et al. *JAMA Network Open*.

eTable 3. Associations Between Quartile-Based Physical Activity Levels and Risk of Incident All-Cause Dementia Among Early Adult Life, Midlife, and Late-Life Adults

	**Early adult life**		**Midlife**		**Late-life**	
**Physical activity index**	**Hazard ratio[a] (95% confidence interval)**	***P***	**Hazard ratio[a] (95% confidence interval)**	***P***	**Hazard ratio[a] (95% confidence interval)**	***P***
Quartiles						
1st quartile (lowest)	1.00 (Reference)		1.00 (Reference)		1.00 (Reference)	
2nd quartile	1.36 (0.68, 2.71)	0.38	0.95 (0.69, 1.31)	0.76	0.87 (0.61, 1.24)	0.44
3rd quartile	1.13 (0.55, 2.31)	0.74	0.79 (0.57, 1.11)	0.18	0.84 (0.59, 1.19)	0.33
4th quartile (highest)	0.86 (0.40, 1.84)	0.70	0.69 (0.49, 0.96)	0.03	0.65 (0.44, 0.95)	0.03

P = p-value

[a]Adjusted for age, sex, education, body mass index, smoking status, hypertension, diabetes, hyperlipidemia, *APOE* ε4 carrier status

© 2025 Marino FR et al. *JAMA Network Open*.

eTable 4. Associations Between Physical Activity Intensity (Slight, Moderate, Heavy) Levels and Risk of Incident All-Cause Dementia Among Early Adult Life, Midlife, and Late-Life Adults

	Early adult life		Midlife		Late-life	
**Physical activity intensity**	**Hazard ratio[a] (95% confidence interval)**	***P***	**Hazard ratio[a] (95% confidence interval)**	***P***	**Hazard ratio[a] (95% confidence interval)**	***P***
Slight physical activity						
1st quintile (lowest)	1.00 (Reference)		1.00 (Reference)		1.00 (Reference)	
2nd quintile	1.34 (0.59, 3.00)	0.48	1.27 (0.87, 1.87)	0.22	1.38 (0.91, 2.07)	0.12
3rd quintile	1.33 (0.60, 2.96)	0.48	1.25 (0.85, 1.84)	0.26	1.34 (0.89, 2.01)	0.16
4th quintile	1.13 (0.49, 2.60)	0.77	1.23 (0.83, 1.81)	0.30	0.95 (0.62, 1.46)	0.82
5th quintile (highest)	1.02 (0.44, 2.36)	0.97	1.18 (0.80, 1.76)	0.40	0.80 (0.51, 1.26)	0.34
Moderate physical activity						
1st quintile (lowest)	1.00 (Reference)		1.00 (Reference)		1.00 (Reference)	
2nd quintile	0.96 (0.43, 2.14)	0.92	0.92 (0.63, 1.34)	0.66	1.03 (0.68, 1.57)	0.88
3rd quintile	0.70 (0.29, 1.68)	0.42	0.80 (0.54, 1.17)	0.25	0.89 (0.58, 1.37)	0.61
4th quintile	0.64 (0.27, 1.50)	0.30	0.65 (0.44, 0.97)	0.03	0.77 (0.49, 1.22)	0.27
5th quintile (highest)	0.57 (0.23, 1.43)	0.23	0.62 (0.42, 0.92)	0.02	0.76 (0.49, 1.17)	0.21
Heavy physical activity						
1st quintile (lowest)	1.00 (Reference)		1.00 (Reference)		1.00 (Reference)	
2nd quintile	1.66 (0.73, 3.74)	0.22	0.96 (0.66, 1.40)	0.84	1.00 (0.68, 1.47)	0.99
3rd quintile	1.48 (0.67, 3.31)	0.33	0.96 (0.66, 1.38)	0.82	0.86 (0.57, 1.30)	0.48
4th quintile	1.10 (0.48, 2.56)	0.81	0.96 (0.66, 1.39)	0.82	0.83 (0.55, 1.27)	0.39
5th quintile (highest)	0.88	0.80	0.66	0.05	0.78	0.39

© 2025 Marino FR et al. *JAMA Network Open*.

	(0.35, 2.26)		(0.44, 1.00)		(0.52, 1.17)	

P = p-value

[a]Adjusted for age, sex, education, body mass index, smoking status, hypertension, diabetes, hyperlipidemia, *APOE* ε4 carrier status

© 2025 Marino FR et al. *JAMA Network Open*.

eTable 5. Associations Between Physical Activity and Risk of Incident Alzheimer's Disease Dementia Among Early Adult Life, Midlife, and Late-Life Adults

	Early adult life (1,526 participants/35 incident AD dementia cases)				Midlife (1,943 participants/178 incident AD dementia cases)				Late-life (885 participants/156 incident AD dementia cases)			
	Crude model		Adjusted model[a]		Crude model		Adjusted model[a]		Crude model		Adjusted model[a]	
**Physical activity index**	**HR (95% CI)**	***P***	**HR (95% CI)**	***P***	**HR (95% CI)**	***P***	**HR (95% CI)**	***P***	**HR (95% CI)**	***P***	**HR (95% CI)**	***P***
Quintiles												
1st quintile (lowest)	1.00 (Reference)		1.00 (Reference)		1.00 (Reference)		1.00 (Reference)		1.00 (Reference)		1.00 (Reference)	
2nd quintile	1.21 (0.50, 2.92)	0.67	1.07 (0.40, 2.88)	0.90	0.87 (0.57, 1.34)	0.53	0.99 (0.60, 1.63)	0.97	1.03 (0.65, 1.62)	0.91	1.19 (0.71, 1.99)	0.50
3rd quintile	0.68 (0.24, 1.92)	0.47	0.82 (0.23, 2.93)	0.76	0.80 (0.52, 1.24)	0.32	0.80 (0.49, 1.30)	0.38	0.88 (0.54, 1.42)	0.59	1.11 (0.70, 1.77)	0.66
4th quintile	0.55 (0.19, 1.65)	0.29	0.64 (0.19, 2.17)	0.48	0.66 (0.41, 1.05)	0.08	0.65 (0.38, 1.11)	0.11	0.63 (0.38, 1.05)	0.08	0.74 (0.43, 1.28)	0.28
5th quintile (highest)	0.44 (0.14, 1.43)	0.17	0.43 (0.11, 1.70)	0.23	0.62 (0.49, 1.00)	0.05	0.55 (0.33, 0.94)	0.03	0.57 (0.33, 0.99)	0.05	0.53 (0.29, 0.95)	0.03

AD = Alzheimer's disease; HR = hazard ratio; *P* = p-value; Q1 = 1st quintile; Q2 = 2nd quintile; Q3 = 3rd quintile; Q4 = 4th quintile; Q5 = 5th quintile

[a]Adjusted for age, sex, education, body mass index, smoking status, hypertension, diabetes, hyperlipidemia, *APOE* ε4 carrier status

© 2025 Marino FR et al. *JAMA Network Open.*

eTable 6. Associations Between Physical Activity and Risk of Incident Alzheimer's Disease Dementia Among Early Adult Life, Midlife, and Late-Life Adults by *APOE* ε4 Carrier Status

**ε4 carrier only**												
	Early adult life				Midlife				Late-life			
	Crude model		Adjusted model[a]		Crude model		Adjusted model[a]		Crude model		Adjusted model[a]	
**Physical activity index**	**HR (95% CI)**	***P***	**HR (95% CI)**	***P***	**HR (95% CI)**	***P***	**HR (95% CI)**	***P***	**HR (95% CI)**	***P***	**HR (95% CI)**	***P***
Quintiles												
1st quintile (lowest)	1.00 (Reference)		1.00 (Reference)		1.00 (Reference)		1.00 (Reference)		1.00 (Reference)		1.00 (Reference)	
2nd quintile	2.01 (0.70, 5.80)	0.20	2.30 (0.78, 6.78)	0.13	1.85 (0.87, 3.92)	0.11	1.56 (0.61, 3.98)	0.35	1.16 (0.43, 3.14)	0.76	0.93 (0.35, 2.45)	0.89
3rd quintile	0.90 (0.24, 3.45)	0.88	0.90 (0.23, 3.60)	0.89	1.38 (0.63, 3.04)	0.42	1.40 (0.56, 3.49)	0.47	0.91 (0.35, 2.36)	0.84	0.83 (0.25, 2.79)	0.77
4th quintile	0.75 (0.20, 2.82)	0.67	0.69 (0.15, 3.17)	0.64	1.22 (0.55, 2.69)	0.63	1.23 (0.54, 2.83)	0.62	0.79 (0.26, 2.45)	0.69	0.82 (0.28, 2.40)	0.72
5th quintile (highest)	0.38 (0.08, 1.85)	0.23	0.35 (0.07, 1.82)	0.21	0.92 (0.38, 2.22)	0.85	0.76 (0.28, 2.08)	0.59	0.79 (0.26, 2.38)	0.68	0.72 (0.23, 2.78)	0.58
**Non-ε4 carrier only**												
	Early adult life				Midlife				Late-life			
	Crude model		Adjusted model[a]		Crude model		Adjusted model[a]		Crude model		Adjusted model[a]	
**Physical activity index**	**HR (95% CI)**	***P***	**HR (95% CI)**	***P***	**HR (95% CI)**	***P***	**HR (95% CI)**	***P***	**HR (95% CI)**	***P***	**HR (95% CI)**	***P***
Quintiles												
1st quintile (lowest)	1.00 (Reference)		1.00 (Reference)		1.00 (Reference)		1.00 (Reference)		1.00 (Reference)		1.00 (Reference)	
2nd quintile	1.19 (0.27, 5.29)	0.82	1.24 (0.28, 5.53)	0.78	0.60 (0.34, 1.05)	0.07	0.57 (0.31, 1.06)	0.07	0.90 (0.52, 1.53)	0.69	1.00 (0.57, 1.74)	0.99
3rd quintile	0.96 (0.20, 4.73)	0.96	1.03 (0.19, 5.52)	0.97	0.56 (0.32, 0.99)	0.05	0.57 (0.31, 1.03)	0.06	0.58 (0.33, 1.03)	0.06	0.69 (0.38, 1.28)	0.24
4th quintile	0.93 (0.19, 4.58)	0.92	0.89 (0.18, 4.58)	0.89	0.53 (0.30, 0.95)	0.03	0.45 (0.24, 0.85)	0.01	0.48 (0.26, 0.90)	0.02	0.68 (0.35, 1.30)	0.24
5th quintile (highest)	0.63 (0.11, 3.79)	0.61	0.69 (0.11, 4.15)	0.68	0.46 (0.25, 0.84)	0.01	0.35 (0.18, 0.70)	<0.01	0.39 (0.19, 0.79)	0.01	0.43 (0.21, 0.89)	0.02
$P_{interaction}$[b]	0.79				0.01				0.20			

© 2025 Marino FR et al. *JAMA Network Open*.

AD = Alzheimer's disease; HR = hazard ratio; *P* = p-value; Q1 = 1st quintile; Q2 = 2nd quintile; Q3 = 3rd quintile; Q4 = 4th quintile; Q5 = 5th quintile

[a]Adjusted for age, sex, education, body mass index, smoking status, hypertension, diabetes, and hyperlipidemia

[b]P-value for interaction between physical activity and *APOE* ε4 carrier status reflects physical activity operationalized as a dichotomous variable (higher physical activity index (Q2-Q5) versus low physical activity index (Q1)), and is based on the adjusted model

© 2025 Marino FR et al. *JAMA Network Open*.
