# Supplementary material for: Physical Activity Over the Adult Life Course and Risk of Dementia in the Framingham Heart Study
Source: JAMA Netw Open. 2025 Nov 19;8(11):e2544439. doi: 10.1001/jamanetworkopen.2025.44439 (PMC12631490; doi:10.1001/jamanetworkopen.2025.44439)
Supplement: Supplement 2. — Data Sharing Statement [file jamanetwopen-e2544439-s002.pdf]

Data Sharing Statement

Marino. Physical Activity Over the Adult Life Course and Risk of Dementia in the Framingham Heart Study. *JAMA Netw Open*. Published November 19, 2025. doi:10.1001/jamanetworkopen.2025.44439

Data
Data available: No

Additional Information
Explanation for why data not available: The datasets generated and analyzed for this study could be shared on reasonable request after approval of a Framingham Heart Study research application.
